# Relationship between Microstructure and Properties of Cu-Cr-Ag-(Ce) Alloy Using Microscopic Investigation

**DOI:** 10.1155/2017/4646581

**Published:** 2017-07-10

**Authors:** Huiming Chen, Dawei Yuan, Shanjiang Wu, Hang Wang, Weibin Xie, Bin Yang

**Affiliations:** ^1^Institute of Engineering Research, Jiangxi University of Science and Technology, Ganzhou, China; ^2^School of Materials Science and Engineering, Jiangxi University of Science and Technology, Ganzhou, China

## Abstract

Microstructure, precipitation hardening response, and mechanical and physical properties of Cu-Cr-Ag alloy and Cu-Cr-Ag-Ce alloy have been investigated using transmission electron microscopy, scanning electron microscope, optical microscope, electrical conductivity analysis, and tensile test. The influence of element Ce on the matrix refinement, impurity removal, and precipitation in the Cu-Cr-Ag alloys has been analyzed. The experimental results show that the strength and electrical conductivity of Ce containing alloys are greater than those of Ce-free alloys after each processing step. Improvement of strength and electrical conductivity of the Cu-Cr-Ag alloy by adding Ce element is attributed to removing oxygen and sulfur from as-cast alloy.

## 1. Introduction

With the rapid development of high-speed electrified railway industry, there is urgent need to enhance the qualities of contact wire materials, including high conductivity, high strength, and moderate plasticity [1, 2]. The Cu-based alloys with low-chromium and low-silver are attractive candidates for contact wire materials due to the excellent performance of mechanical strength and electrical conductivity [3]. The excellent strength of the alloy is attributed to dispersion strengthening of Cr precipitates; and the high electrical conductivity of the alloy is due to the very low solubility of chromium and silver in copper solution [4].

Rare earth (RE) elements usually show many beneficial effects on microstructure and mechanical properties in conventional copper alloys [5, 6]. Use of RE as microalloying elements in Cu has been studied for several years. Guo et al. [7] and Mao et al. [8] found that RE easily reacted with harmful elements to form intermetallic compounds, which resulted in purification of the alloy melts. Zhang et al. [9, 10] reported that RE elements have strong grain refining effects on the as-cast alloy by retarding the grain boundary motion in the process of grain growth, which can optimize the hot workability of the copper alloy. Trace addition of RE could effectively inhibit the growth of precipitates because RE atoms with large atomic radius distributed along the grain boundaries and dislocations would block the short range diffusion tunnel of the solute atoms [11, 12].

In the present work, trace Ce element was added to the Cu-Cr-Ag alloy; and microstructures and mechanical and physical properties of the Cu-Cr-Ag alloys with and without Ce were investigated. Influence of Ce element on the microstructure and properties was determined using microscopic investigation.

## 2. Experimental

The experimental alloys were melted in medium frequency induction furnace (ZP-45, Jiangyin Ruibang) using cathode copper, high purity silver, Cu-10 wt% Cr master-alloy, and pure cerium and then cast into iron mold with Φ80 mm × 200 mm dimensions. The mean compositions of the experimental alloys were given in Table 1. The real compositions were determined by an inductively coupled plasma emission spectrometer (ICP, IRIS Intrepid II, Thermo Fisher Scientific). The ingots were homogenized at 920°C for 1 h and then hot extruded into rod of Φ12 mm. Subsequently, the diameter of rods was reduced to Φ4.5 mm by multipass cold drawing. The cold drawn rods were solution treated at 950°C for 1 h and quenched in water to obtain supersaturated solid solution. Finally, the solution treated samples were annealed between 400°C and 550°C for durations between 30 min and 240 min.

A standard metallographic technique was used to prepare the samples. This technique included wet grinding, prepolishing, “Minimet” polishing, and etching with a solution containing 20 mL HNO_3_ in 80 mL water. An optical microscope (OM, BMM-90AE, Shanghai Bimu) and a scanning electron microscopy (SEM, MLA650F, FEI) were used to observe the microstructure of the samples. Grain size was measured through quantitative image analysis, based on American Society for Testing and Materials (ASTM) standard test method E112. The morphology and elemental distribution of the Cu-Cr-Ag alloy were examined by scanning electron microscopes (SEM, MIRA3 LMH, TESCAN-ORSAY HOLDING a.s.) equipped with energy-disperse spectrometer (EDS, 9806A-1UPS-SN, Thermo Fisher Scientific) attachment.

The specimens for TEM observation were sliced from bulk materials, ground into thin foils with 60 *μ*m thickness, and then punched into circle foils with a diameter of 3 mm. The specimen foils were electropolished at −35°C at a voltage of 40 V in a twin-jet electropolisher. The polishing solution was 30 vol% nitric acid and 70 vol% methanol. Subsequently, the foils were examined in a transmission electron microscopy (TEM, Tecnai-G2-F20, FEI) operating at 200 kV.

In addition, tensile tests were performed at room temperature on a universal testing machine (UTM5105X, Shenzhen Sunthink). The mechanical results reported in this study are the mean values of at least five specimens. Electrical conductivity was measured at room temperature by a direct current resistance tester (SB2230, Shanghai Guosheng). The conductivity was measured and evaluated according to the international annealing copper standard (IACS, 100% IACS = 1.7241 *μ*Ω·cm).

## 3. Results 

### 3.1. Alloy Composition

Table 2 shows the real chemical compositions of the two alloys by ICP. Comparing Table 2 with Table 1, it is clear that the loss of Ce is high during melting of Cu-Cr-Ag alloy. It is indicated that the Ce element is easy to be consumed for reacting with other elements due to its active chemical properties. In addition, the Ce containing alloy has oxygen and sulfur contents of 0.0024 and 0.001 wt.%, respectively. The contents of elemental S and O are decreased significantly by adding elemental Ce. It is revealed that the oxygen and sulfur removing reaction takes place through the addition of Ce element, which could effectively purify the melt.

### 3.2. Microstructure

**Figure 1:**
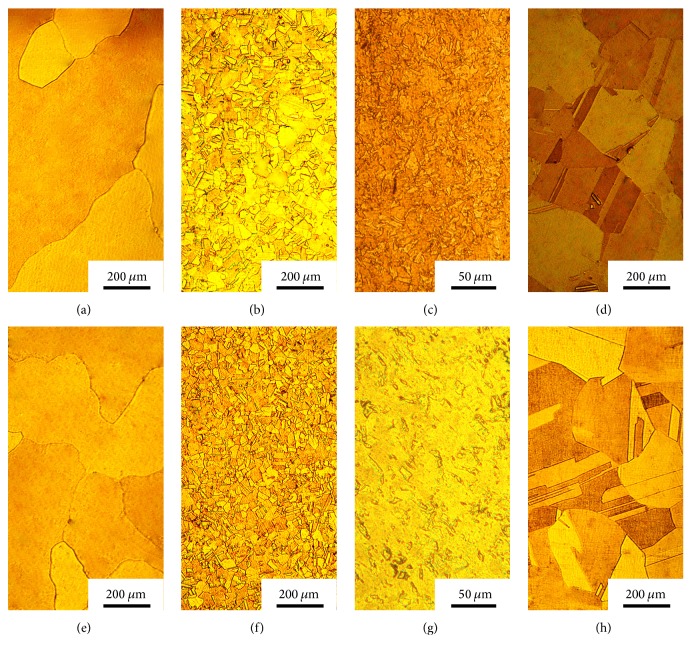
Optical micrograph of the Cu-Cr-Ag alloy (a–d) and Cu-Cr-Ag-Ce alloy (e–h) after different treatment: (a, e) as-cast alloy; (b, f) hot extruded alloy; (c, g) cold drawn alloy; (d, h) solution treated alloy.

The microstructures of the Cu-Cr-Ag alloys under different treatment conditions are shown in Figure 1. The large as-cast grains of Cu-Cr-Ag alloy, about 460 *μ*m mean diameter, are observed in Figure 1(a). After hot extruding and cold drawing, the grains are obviously refined as shown in Figures 1(b) and 1(c). The grains become equiaxed after solid solution treatment due to the recrystallization (Figure 1(d)). The evolution of microstructure in Cu-Cr-Ag-Ce alloy is similar to that in Cu-Cr-Ag alloy (Figures 1(e)–1(h)).

Despite the same melting and casting processes, the as-cast Cu-Cr-Ag alloys with or without Ce show different microstructures. The average grain diameter of as-cast Cu-Cr-Ag-Ce alloy, about 340 um, is significantly smaller than that of as-cast Cu-Cr-Ag alloy. It is indicated that the Ce addition could refine the microstructure of the as-cast Cu-Cr-Ag alloy. On the one hand, Ce could react with S, O, and other elements forming high melting point compounds, and these compounds can increase the nucleation rate of grains. On the other hand, the corresponding atoms transport from the liquid phase to the solid phase is hindered, since Ce atoms segregate on the liquid-solid two-phase interface during solidification. Consequently, the growth of grains was prevented, and the grains became finer. However, the difference of grain sizes in the two alloys is reduced after hot extruding, cold drawing, and solution treatment. The average grain size of solution treated Cu-Cr-Ag-Ce alloy (175 *μ*m in diameter) is very similar to that of solution treated Cu-Cr-Ag alloy (190 *μ*m diameter). The Cu-Cr-Ag alloys with and without Ce element after solution treating exhibit similar morphology like grain shape and size, and the grain refining effect of Ce addition in Cu-Cr-Ag alloy is not obvious after forming and heat treatment.

**Figure 2:**
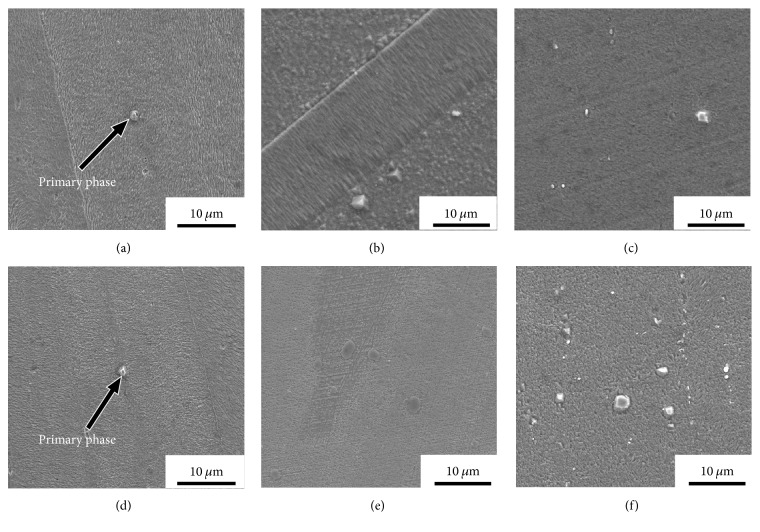
SEM micrograph of the Cu-Cr-Ag alloy (a–c) and Cu-Cr-Ag-Ce alloy (d–f) after different treatment: (a, d) as-cast alloy; (b, e) solution treated alloy; (c, f) aged alloy at 500°C for 120 min.

**Figure 3:**
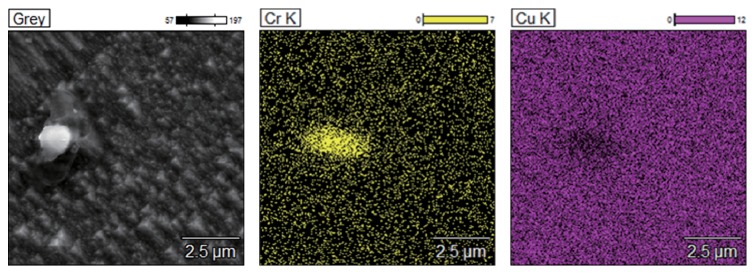
EDS mapping result of solution treated Cu-Cr-Ag alloy.

The SEM micrographs of the Cu-Cr-Ag alloys under different treatment conditions are shown in Figure 2. It is revealed that these images of the Cu-Cr-Ag alloy and the Cu-Cr-Ag-Ce alloy are similar. The coarse primary phases are observed in the matrix of the as-cast alloys (Figures 2(a) and 2(d)). The edges of primary phases are smoothed out during solution treating, which revealed that the primary phases can dissolve into the matrix at a high temperature ~950°C (Figures 2(b) and 2(e)). However, the primary phases cannot entirely dissolve into the matrix after solution treating. The EDS investigations confirm that the undissolved primary phases in Cu-Cr-Ag alloy after solution treating are Cr particles (Figure 3). It is indicated that Cr particles tend to form during the solidification. In addition, the dispersed Cr precipitates cannot be observed in the SEM micrographs of the peak-aging alloys (Figures 2(c) and 2(f)). In order to observe the transformation process during aging, the detailed microstructural analysis was performed using TEM.

**Figure 4:**
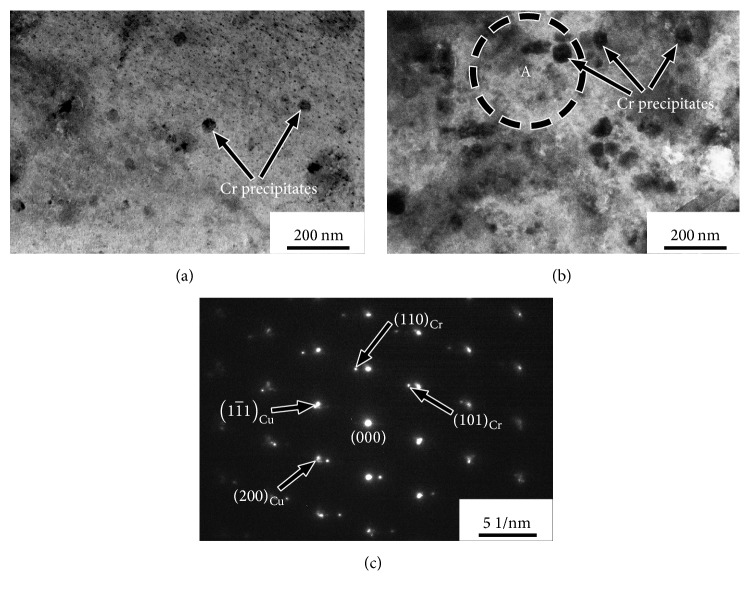
Bright field TEM microstructure of aged Cu-0.27Cr-0.083Ag-0.015Ce alloy: (a) aged alloy for 30 min at 500°C; (b) aged alloy for 120 min at 500°C; (c) the SAED pattern of area A.

Figure 4 is the bright field TEM image showing the microstructure of the Cu-Cr-Ag-Ce alloy aged at 500°C for 30 min and 120 min. The TEM micrograph of aging-treated condition shows the presence of some fine precipitates in the matrix. Using selected area electron diffraction (SAED) technique (Figure 4(c)), the large particle with a body-centered cubic structure (lattice parameter is about 0.292 nm) could be identified as Cr precipitates. The average size of Cr particles increased gradually with the increase of the aging time. The spherical precipitate size of peak-aged Cu-Cr-Ag-Ce alloy is about 55–70 nm aged at 500°C for 120 min (Figure 4(b)). Only spherical Cr particles are found within the grains in aged Cu-Cr-Ag-Ce alloy, which is similar to that observed in Cu-Cr-Ag alloys and Cu-Cr alloys [4, 13, 14]. It is indicated that the aging process is practically growing process of the Cr precipitate particles in Cu-Cr-Ag-Ce alloy.

### 3.3. Mechanical and Physical Properties

**Figure 5:**
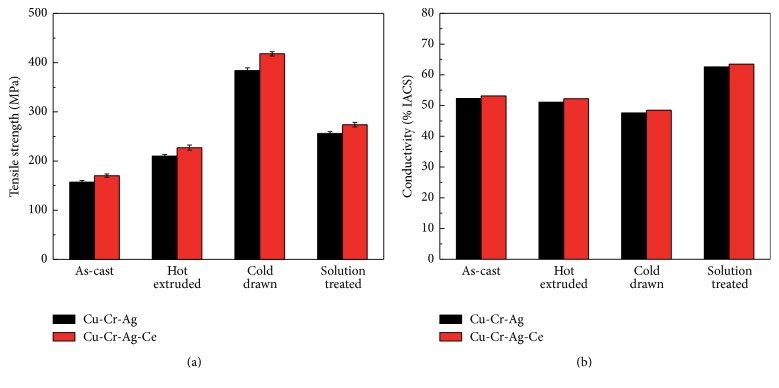
Mechanical and physical properties of Cu-Cr-Ag alloys after different treatments: (a) strength; (b) electrical conductivity.

The strengths and electrical conductivities of Cu-0.29Cr-0.082Ag alloy and Cu-0.27Cr-0.083Ag-0.015Ce alloy under various treatment conditions are shown in Figure 5. It can be seen that the strength of the Cu-0.29Cr-0.082Ag alloy ingot is increased due to the effect of work hardening after hot extruding and cold drawing. And the strength of this alloy is decreased due to recrystallization during solution treatment (Figure 5(a)). The variation trend of tensile strength in Cu-0.29Cr-0.082Ag alloy and Cu-0.27Cr-0.083Ag-0.015Ce alloy is consistent.

The lattice distortion or internal fault structure caused by deformation would hinder the migration of the electron. Thereby, the electrical conductivity decreases due to the introduction of defects like dislocations after hot extruding and cold drawing (Figure 5(b)). The dislocations and grain boundaries of the matrix become less due to recrystallization after solution treatment; therefore the electrical conductivity is 47.5 ± 0.1% IACS after solution treatment in the Cu-Cr-Ag alloy (the errors of electrical conductivity are small, and error bars are neglected in figure of electrical conductivity). In addition, the strength and electrical conductivity of Ce containing alloy are always higher than Ce-free alloy under all treatment processes.

**Figure 6:**
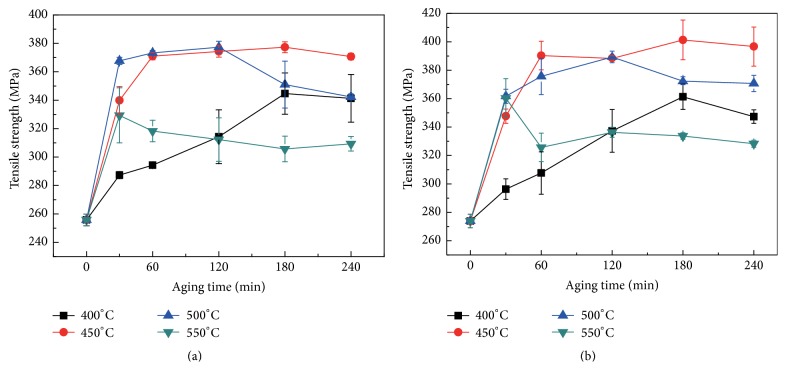
Tensile strength of aged experimental alloys: (a) Cu-0.29Cr-0.082Ag alloy and (b) Cu-0.27Cr-0.083Ag-0.015Ce alloy.

The variation of tensile strength with aging time and temperature for Cu-Cr-Ag alloys is shown in Figure 6. It can be observed that the strength of Cu-Cr-Ag alloys is increased rapidly with time in the initial stage and then gradually decreases. The strength of Cu-Cr-Ag-Ce alloy is improved with increasing Cr particle size during initial period of aging. The strength is gradually decreased by a progressive coarsening of Cr precipitates, which caused the overaging in the later stage of aging treatment process. It is indicated that the size and distribution of the precipitates exert a strengthening effect on mechanical properties. The maximum strengths of Cu-Cr-Ag alloys can reach a maximum at 500°C. The result of mechanical properties change during aging treatment of Cu-Cr-Ag-Ce alloy is consistent with that in Cu-Cr-Ag alloy. However, the maximum strength of Ce containing alloy is always higher than Ce-free alloy at the same temperature. The maximum strength of Cu-Cr-Ag alloy and Cu-Cr-Ag-Ce alloy aging at 500°C is 379 MPa and 388 MPa, respectively.

**Figure 7:**
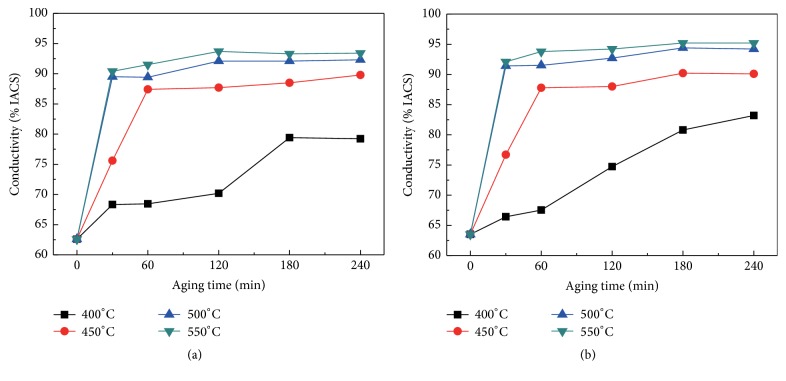
Electrical conductivity of aged experimental alloys: (a) Cu-0.29Cr-0.082Ag; (b) Cu-0.27Cr-0.083Ag-0.015Ce.

The relation of the electrical conductivity and the aging conditions is plotted in Figure 7. It can be observed that the conductivity of the Cu-Cr-Ag alloys was improved with the increase of the aging time and temperature. The maximum electrical conductivity of Cu-Cr-Ag alloy and Cu-Cr-Ag-Ce alloy aging at 550°C is 95.0 ± 0.2% IACS and 93.3 ± 0.1% IACS, respectively. The electrical conductivity increases sharply and then tends to be stable during aging treatment. The dissolved solutes in pure copper will rapidly reduce due to Cr element precipitate from the Cu-rich matrix, which induces the increase of electrical conductivity at the initial stage of aging. It is considered that the Cr element precipitates from supersaturated solid solution through diffusion of the solute atoms with the aid of the vacancies. Therefore, the Cr precipitation becomes slower due to the decrease of supersaturated vacancies in the later stage of aging treatment process. The precipitation transformation is kinetically controlled by diffusion. Therefore, the increase in conductivity at the initial stage is faster with increasing aging temperature, since the precipitation rate becomes higher at a higher temperature. In addition, the changing tendency of electrical conductivity of the Cu-Cr-Ag alloy and Cu-Cr-Ag-Ce alloy is consistent.

## 4. Discussion

The present study shows that the addition of Ce affected the physical and mechanical properties in Cu-Cr-Ag alloy. It can be found that the conductivity of Ce containing alloy is always 1-2% IACS higher than Ce-free alloy, and the strength of Ce-free alloy is always 15–30 MPa higher than that of Ce-free alloy during forming and heat treating process. It is indicated that Ce addition could slightly improve the strength and electrical conductivity of Cu-Cr-Ag alloy.

The addition of Ce element can refine the microstructure of the as-cast Cu-Cr-Ag alloy. Nevertheless, the plastic deformation and heat treatment have significant effect on morphology like grain shape and size in Cu-Cr-Ag alloys. There is no significant difference in the grain size between Cu-Cr-Ag alloy and Cu-Cr-Ag-Ce alloy during forming and heat treating process. It is revealed that the difference of properties between the two alloys is independent of grain size. The grain refining effect of Ce element on as-cast Cu-Cr-Ag alloy is not the main factor for the improvement of physical and mechanical properties. In addition, the differences of properties between the two alloys are stable during the whole process. It is indicated that the higher conductivity and strength in Ce containing alloy may be caused by the alloy composition.

The present study also shows that the sulfur and oxygen contents in Cu-Cr-Ag are reduced with the addition of Ce (Table 2). It is revealed that the S and O contents in Ce containing alloy are lower than that in the Ce-free alloy. The addition of Ce could deprive O and S elements from Cu-Cr-Ag alloy. It is reported that Ce element is an element with high chemical activity, and the added Ce could react preferentially with O and S by forming high melting point and stable compounds during casting. The CeS, Ce_2_O_3_, and CeO_2_ have lower densities, and it is easy to be removed with slag. The obstacles for the electron migration thus become less because the magnitude of S and O element in grain interiors and at grain boundaries is decreased. Therefore, the conductivity of Ce containing alloy is higher. In addition, the sulfur and oxygen elements are harmful for strength and plasticity [15, 16]. For example, the embrittlement of copper is caused by sulfur impurity located at grain boundaries [16]. Therefore, the strength is increased with decreasing the oxygen and sulfur content in Cu-Cr-Ag alloy. It is revealed that the impurity removal effect of Ce element on Cu-Cr-Ag alloys is the main factor for the improvement of physical and mechanical properties during deformation and heat treatment.

Comparing the mechanical and physical properties of the Cu-Cr-Ag alloy and Cu-Cr-Ag-Ce alloy during aging treatment, it is observed that the Ce addition has little effect on microstructure evolution and mechanical and physical properties. The morphology and growth regularity of Cr precipitates in Cu-Cr-Ag-Ce alloy are consistent with those in Cu-Cr-Ag alloy. The changing tendency of strength and electrical conductivity in the Cu-Cr-Ag alloy and Cu-Cr-Ag-Ce alloy is similar. The element Ce will be distributed on both grain boundaries and defects of Cu-Cr-Ag alloy during solidification, due to the lower melting point of Ce and its large atomic radius [12]. However, the grain boundary migration and the reduction in internal crystal defect occurred by recrystallization, which will result in decreasing of the amount of elemental Ce on the grain boundaries and defects [17]. Therefore, the Ce cannot effectively inhibit the growth of precipitates and refine the precipitates. There is a little effect on aging precipitation behavior by adding Ce element in the Cu-Cr-Ag alloy.

As discussed above, the main role of the added Ce during deformation and heat treatment in Cu-Cr-Ag alloy will not refine grains and precipitates but purify the melts. It should also be considered that the elimination of the impurities such as O and S could effectively increase strength and electrical conductivity of Cu-Cr-Ag alloy.

## 5. Conclusion

The effect of Ce element on microstructure and mechanical and physical properties of Cu-Cr-Ag alloy was investigated by optical microscope, scanning electron microscope, transmission electron microscopy, and mechanical tests. The role of Ce on the mechanical and physical properties was discussed. The following conclusions can be drawn from this work,

1. The addition of Ce element in Cu-Cr-Ag alloy during melting can bring about deprivation of harmful elements such as S and O from melt and also refine the as-cast microstructure.
2. The recrystallization will occur during solution treating due to severe plastic deformation, which results in the fact that the effects of Ce element on grain refining and precipitate refining are not apparent. The improvement of physical and mechanical properties in the Ce containing alloy during deformation and heat treatment is manly attributed to purification of Cu-Cr-Ag alloy by adding Ce during melting.

## Figures and Tables

**Table 1:** Mean composition for the experimental alloys.

Alloy	Mean composition (wt.%)			
	Cr	Ag	Ce	Cu
Cu-Cr-Ag	0.3	0.1	—	Bal.
Cu-Cr-Ag-Ce	0.3	0.1	0.025	Bal.

**Table 2:** Real compositions for the experimental alloys.

Alloy	Real composition (wt.%)					
	Cr	Ag	Ce	O	S	Cu
Cu-Cr-Ag	0.29	0.082	—	0.0033	0.0032	Bal.
Cu-Cr-Ag-Ce	0.27	0.083	0.015	0.0024	0.001	Bal.
